# Modulation of gut microbiota and metabolic profiles by Astragaloside IV in a chronic enteritis model

**DOI:** 10.3389/fcimb.2026.1645113

**Published:** 2026-03-11

**Authors:** Jian-xia Liu, Yue-hong Xu, Si-di Li, Yong-fang Zhang, Jia-chuan Yang, Run-li He

**Affiliations:** 1 College of Agriculture and Life Sciences, Shanxi Datong University, Datong, Shanxi, China; 2 Southwest Hospital, Army Medical University (Third Military Medical University), Chongqing, China; 3 College of Traditional Chinese Medicine and Food Engineering, Shanxi University of Chinese Medicine, Taiyuan, Shanxi, China

**Keywords:** Astragaloside IV, chronic intestinal inflammatory, gut-, metabolome, microbiota

## Abstract

Chronic intestinal inflammatory diseases like ulcerative colitis severely impact millions globally. Current treatments using drugs manage inflammation short-term but have significant side effects and don’t address underlying issues like mucosal barrier repair. New treatments are needed, and Chinese herbal medicine is gaining attention for its low cost and side effects. This study used a DSS-induced chronic enteritis animal model and treated it with Astragaloside IV(AS-IV), a traditional Chinese medicine. 16S sequencing and LC-MS analysis showed that AS-IV significantly reduces DSS-induced inflammation. This encompasses an enhancement in body weight, a reduction in the bleeding index, diarrhea index, and disease activity index (DAI). Furthermore, AS-IV has the capacity to modulate gut microbiota diversity, augment the abundance of beneficial bacteria, diminish the prevalence of harmful bacteria, and mitigate disease progression through alterations in metabolic profiles. Integrated omics analysis has revealed that AS-IV can attenuate disease progression by modulating the biosynthesis of primary bile acids. In summary, this study showed that AS-IV significantly protects against chronic enteritis, as demonstrated by a DSS-induced colitis model. It explored the mechanisms involving gut microbiota and its metabolites, highlighting a new understanding of AS-IV’s pharmacological effects and the crucial role of gut microbiota in enhancing the anti-inflammatory properties of traditional Chinese medicine monomers.

## 1 Introduction

Chronic colitis, a form of inflammatory bowel disease (IBD), poses a significant global health issue, marked by recurring colon inflammation and immune system dysfunction. Over 3 million people in Europe and North America have IBD, with increasing cases in newly industrialized countries due to Westernized diets and lifestyles (Kaplan, 2015). The exact cause of IBD is unclear, but it involves genetics, environment, and host-microbiota interactions (Zhang et al., 2021). Treatments like 5-aminosalicylates, corticosteroids, and TNF-α monoclonal antibodies aim to reduce immune activity but often offer only temporary relief and fail to address microbial imbalances. Long-term use of these therapies can lead to serious side effects, such as infections and metabolic issues, and may lose effectiveness over time (Kvasnovsky et al., 2015; Stojanov and Berlec, 2024).

The gut microbiota, a complex community of microorganisms, significantly impacts colonic health through interactions with host cells via metabolites (Lavelle and Sokol, 2020). In a healthy gut, beneficial bacteria ferment dietary fibers into short-chain fatty acids (SCFAs) like acetate, propionate, and butyrate, which support colon cells, enhance tight junctions, and promote regulatory T-cell development (Blaak et al., 2020). In contrast, colitis-related dysbiosis involves a reduction in SCFA-producing bacteria, an increase in pro-inflammatory *Proteobacteria*, and disrupted metabolism of bile acids and other compounds (Haase et al., 2020). These changes weaken the gut barrier, activate inflammatory pathways, and shift immune responses towards Th1/Th17 (Cui et al., 2018). Advances in multi-omics have highlighted microbial metabolites as key links between microbiota and host health, making them potential therapeutic targets.

In line with the growing interest in plant-derived prebiotics, such as silver fir wood extract and cocoa polyphenols which have demonstrated microbiota-modulating properties, we focused on AS-IV, a bioactive saponin from the traditional herb Astragalus membranaceus (Sorrenti et al., 2020; Stojanov et al., 2021). AS-IV a cycloartane-type triterpenoid saponin extracted from the traditional medicinal herb *Astragalus membranaceus*, has attracted significant scholarly interest due to its multifaceted anti-inflammatory, antioxidant, and immunomodulatory properties (Liang Y. et al., 2023). Preclinical investigations have demonstrated its effectiveness in mitigating dextran sulfate sodium (DSS)-induced colitis by downregulating pro-inflammatory cytokines (IL-6, TNF-α, IL-1β) and upregulating antioxidant enzymes (SOD, GPx) (Tian et al., 2021; Liang J. et al., 2023; Zhang et al., 2024). Notably, emerging evidence indicates that AS-IV may partially exert these effects through modulation of the gut microbiota. For example, administration of AS-IV in murine models has been shown to increase the abundance of *Akkermansia muciniphila*, a mucin-degrading bacterium linked to enhanced gut barrier function and anti-inflammatory signaling (Lyu et al., 2024). Nonetheless, several critical knowledge gaps remain: (1) In what ways does AS-IV alter the microbial community structure and function within the context of chronic colitis? (2) Which metabolites derived from the microbiota mediate its therapeutic effects?

Although previous studies have strongly demonstrated the potential of AS-IV in alleviating intestinal inflammation, significant gaps remain in the current understanding (Li Z. et al., 2023; Gong et al., 2024). Firstly, most research has focused on acute or short-term colitis models, while the efficacy of AS-IV in chronic and relapsing enteritis models, which more closely mimic human disease characteristics, remains unclear. Secondly, existing studies predominantly concentrate on the direct anti-inflammatory or antioxidant effects of AS-IV; however, whether and how it exerts long-term therapeutic benefits by modulating the complex “host-gut microbiota-metabolite” interaction network lacks systematic and integrated analysis.

This study hypothesizes that AS-IV alleviates chronic colitis by orchestrating a synergistic restoration of gut microbial ecology and associated metabolic networks, thereby re-establishing host-microbiota mutualism. Employing a DSS-induced chronic colitis model, we integrate 16S rRNA sequencing, untargeted metabolomics to systematically map AS-IV’s impact on microbial diversity, taxonomic composition, and metabolic pathways.

## 2 Materials and methods

### 2.1 Mice

Thirty male C57BL/6Cnc mice, aged 8 weeks and weighing between 18 and 22 grams, were procured from Cyagen, Suzhou, China. These mice were housed under specific pathogen-free conditions, maintained on a 12-hour light/dark cycle, at a temperature of 21 °C ± 2 °C, and a relative humidity of 45% ± 10%. The mice were fed freely during the entire study, and there was no difference in food consumption between the different groups. All animal experiments were conducted in accordance with the guidelines of the Institutional Animal Care and Use Committee of Shanxi Datong University(NO.202410261). After 1 week of acclimatization, the mice were randomly separated into 3 groups, with 10 mice/group.

### 2.2 Animal experiments

**Figure 1:**
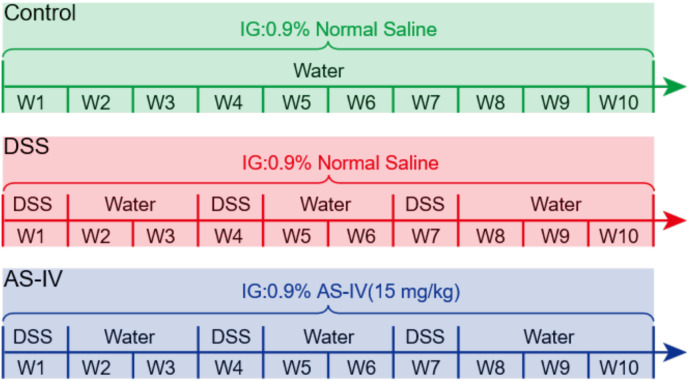
Treatment scheme.

All interventions are depicted in **Figure 1**. During the experimental period, all three groups of mice were administered a 0.9% saline solution via gavage and had ad libitum access to drinking water. The control group received standard sterile water. In contrast, both the model (AS-IV) and intervention groups were initially exposed to a 2% DSS solution for one week, followed by two weeks of normal sterile water; this cycle was repeated three times. Subsequently, the mice were maintained on normal sterile water for an additional week. In the intervention group, AS-IV (Shanghai Aladdin Biochemical Technology Co., Ltd.) was administered orally at the onset of the modeling phase at a dosage of 15 mg/kg, equivalent to 1% of the mice’s body weight. Throughout all experiments, the mice were allowed unrestricted access to food, and no significant differences in food intake were observed among the groups. Throughout the experimental period, the mice were monitored bi-daily for signs of morbidity, and their body weights were systematically recorded. Each mouse was assessed for pathological characteristics, including stool consistency, presence of blood in the stool, and body weight loss. These individual assessments were aggregated to calculate the Disease Activity Index (DAI), following the methodology outlined in prior research (Ma et al., 2023). Diarrhea degree was scored 0 for well-formed pellets, 0.3 for soft pellets, 0.6 for pasty stools, 0.9 for liquid stools. Bleeding degree was scored as 0, when there was no blood; 2, for slight bleeding; 4, for gross bleeding (Wei et al., 2016). Subsequently, all mice were euthanized via an intraperitoneal injection of 0.01–0.02 ml/g of pentobarbital sodium.

### 2.3 Sample collection

All mice were euthanized under general anesthesia. A comprehensive autopsy was conducted to examine the external surface, thoracic cavity, abdominal cavity, and their respective contents. Cecal contents were collected and stored at -80 °C for subsequent 16S rRNA sequencing analysis and non-targeted metabolomics testing. Colon tissue was harvested for RNA isolation and immediately frozen, while the distal colon was preserved for histological examination by fixation in 4% paraformaldehyde. Histopathological analysis of the distal colon was carried out using hematoxylin and eosin (H&E) staining to evaluate the severity of tissue damage induced by colitis.

### 2.4 Microbiota analysis

Cecal contents were collected from each mouse (n=6 per group) for microbiota analysis. Bacterial genomic DNA was extracted from frozen cecal content samples using the QIAamp DNA Stool Mini Kit (Qiagen, Hilden, Germany) following the manufacturer’s instructions. The V3-V4 hypervariable region of the bacterial 16S rRNA gene was amplified by polymerase chain reaction (PCR) using barcoded universal primers 341F (5′-CCTACGGGNGGCWGCAG-3′) and 806R (5′-GGACTACHVGGGTATCTAAT-3′). The purified, quantified, and normalized amplicons were subjected to paired-end sequencing on an Illumina MiSeq PE250 platform (Illumina, San Diego, CA, USA). Sequencing services were provided by Realbio Technology Co., Ltd. (Shanghai, China). The raw paired-end sequencing data were processed using the QIIME2 pipeline (version 2020.11). Briefly, the DADA2 plugin was employed for quality filtering, denoising, merging of paired-end reads, and chimera removal, yielding high-resolution amplicon sequence variants (ASVs). Representative ASV sequences were taxonomically classified against the SILVA database (release 138). Alpha diversity indices (Chao1, Shannon, Simpson) and Beta diversity [based on Bray-Curtis distance, visualized via Principal Coordinates Analysis (PCoA)] were calculated within QIIME2, with statistical comparisons performed between groups (e.g., PERMANOVA). Finally, the Linear Discriminant Analysis Effect Size (LEfSe) method (with an LDA score threshold > 2.0 and p < 0.05) was applied to identify microbial taxa exhibiting significant differential abundance across groups.

### 2.5 Untargeted metabolomics analysis

Cecal contents (25 mg ± 1 mg) were collected and combined with beads and 500 μL of an extraction solution composed of methanol, acetonitrile, and water in a 2:2:1 (v/v) ratio. This extraction solution contained deuterated internal standards. The mixture was subjected to vortexing for 30 seconds. Subsequently, the sample was transferred to a clean Eppendorf tube for centrifugation at 15,000 × g for 20 minutes at 4 °C. The resulting supernatant was then utilized for liquid chromatography-mass spectrometry (LC-MS) analysis. LC-MS/MS analyses were conducted using an ultra-high-performance liquid chromatography (UHPLC) system (Vanquish, Thermo Fisher Scientific) equipped with a Waters ACQUITY UPLC BEH Amide column (2.1 mm × 50 mm, 1.7 μm) and coupled to an Orbitrap Exploris 120 mass spectrometer (Orbitrap MS, Thermo). The mobile phase consisted of 25 mmol/L ammonium acetate and 25 mmol/L ammonia hydroxide in water (pH = 9.75) as solvent A, and acetonitrile as solvent B. The auto-sampler was maintained at a temperature of 4 °C, with an injection volume of 2 μL. The Orbitrap Exploris 120 mass spectrometer was employed for its capability to acquire MS/MS spectra in information-dependent acquisition (IDA) mode, controlled by the Xcalibur software (Thermo). In this mode, the acquisition software continuously evaluates the full-scan MS spectrum. The conditions for the electrospray ionization (ESI) source were configured as follows: a sheath gas flow rate of 50 arbitrary units (Arb), an auxiliary gas flow rate of 15 Arb, and a capillary temperature of 320 °C. The full MS resolution was set at 60,000, while the MS/MS resolution was maintained at 15,000. The collision energy was specified as stepped normalized collision energy (SNCE) at 20/30/40. The spray voltage was adjusted to 3.8 kV for positive ion mode and -3.4 kV for negative ion mode. The raw data were converted to the mzXML format using ProteoWizard. Subsequent data processing, including peak detection, extraction, alignment, and integration, was conducted using an in-house program developed in R, based on the XCMS package. Metabolite identification was facilitated by the R package and BiotreeDB (version 3.0).

### 2.6 Histopathological and immunohistochemical analysis

The colon was fixed in 4% PFA. Paraffin-embedded sections were cut at a thickness of 4 mm and stained with H&E solution. For immunohistochemistry (IHC), paraffin sections were incubated with antibodies specific for Ki-67 antibody (Abcam, cat# ab15580, 1:200 dilution). The specific steps were performed according to previous study.

### 2.7 Statistical analysis

Statistical analysis was performed using GraphPad Prism 10.0 software (GraphPad Software Inc., La Jolla, CA, USA). One-way analysis of variance with Tukey’s multiple comparison correction was used to detect differences among more than two groups. The results are reported as mean ± SD. A P-value < 0.05 was considered significant. Spearman’s correlation analysis was conducted between the bacteria and the UC-related parameters using R Programming Language (V4.0.2, AT&T Bell Laboratories, Auckland, New Zealand).

## 3 Results

### 3.1 AS-IV’s suppression of chronic enteritis

**Figure 2:**
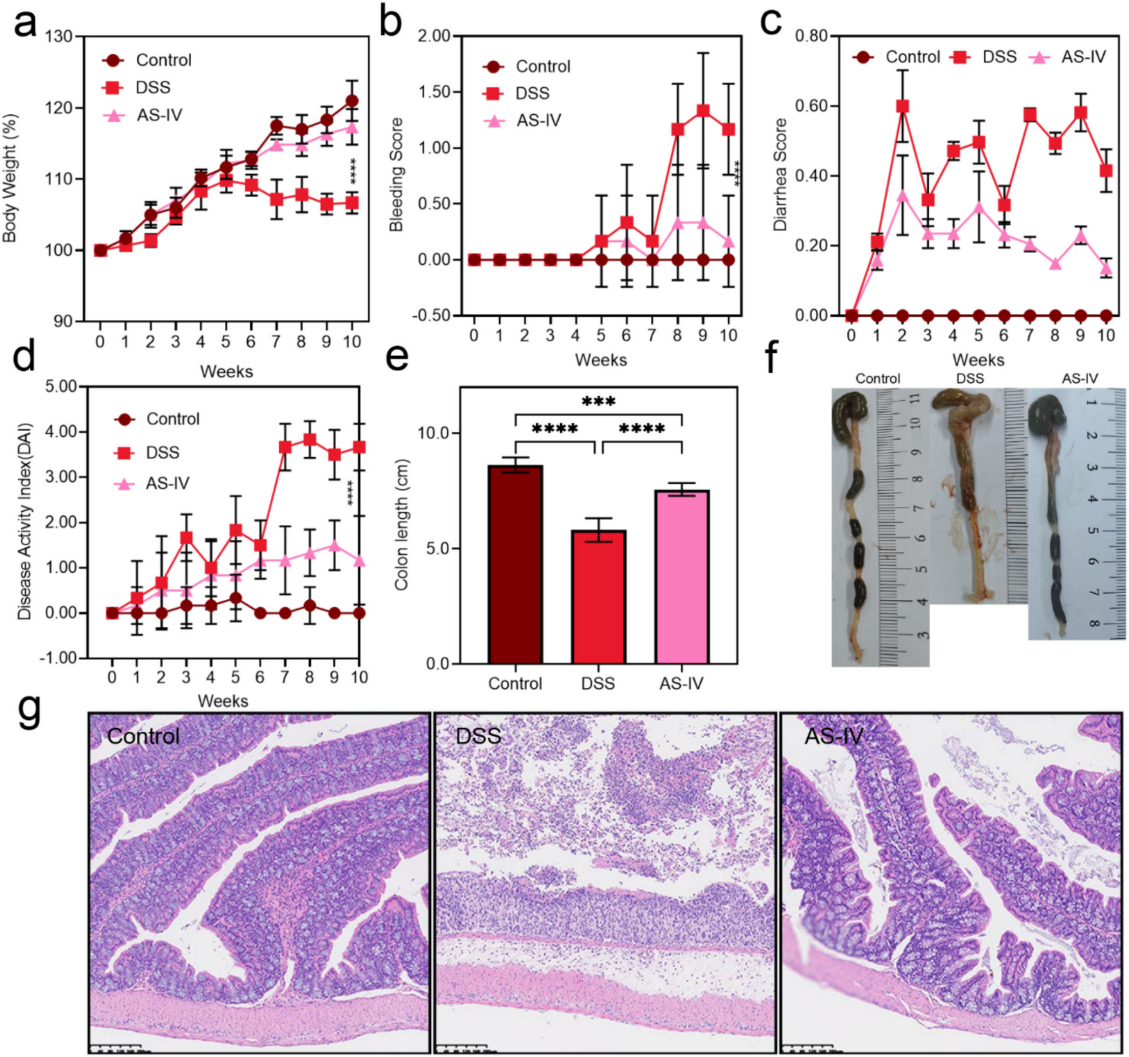
AS-IV administration ameliorated chronic enteritis. **(a)** Body weight change. **(b)** Bleeding score. **(c)** Diarrhea score. **(d)** Disease activity index (DAI) **(e)** Colon length. **(f)** Representative pictures of colon gross appearance. **(g)** Representative microscopic pictures of H&E staining (scale bar 200 μm). **(a–e)** mean values ± SD are presented. P values were calculated using one-way ANOVA with Tukey’s multiple comparison (n = 5 mice per group). ***P < 0.001; ****P < 0.0001.

Initially, regarding alterations in body weight and colon length, the mice in the DSS group exhibited a significant reduction in weight throughout the 10-week experiment. Conversely, in the AS-IV treatment group, this trend of weight loss was notably reversed. Specifically, when compared to the Control group, the DSS group demonstrated a substantial decrease in weight gain, whereas the AS-IV group showed a gradual recovery in weight gain from week 5 to week 10 (**Figure 2a**). Furthermore, improvements were observed in both the bleeding index and diarrhea score. The bleeding index indicated a significant increase in the DSS group by the 10th week, while the AS-IV group experienced a marked alleviation of bloody stool symptoms, as evidenced by a significant decrease in the bleeding index (**Figure 2b**). Mice in the DSS group began experiencing diarrhea from the fifth week, with feces becoming thinner and more difficult to form. However, in the AS-IV treated mice, the diarrhea index significantly decreased, and fecal consistency gradually returned to normal levels (**Figure 2c**). These findings suggest that AS-IV exerts a potent inhibitory effect on DSS-induced intestinal bleeding. The DAI index also shows the same trend (**Figure 2d**). Simultaneously, the colon length in the DSS group was markedly reduced, whereas the colon length in the AS-IV group was significantly restored. This suggests that AS-IV has the potential to effectively mitigate pathological alterations, including colon shortening and weight loss, induced by DSS ((**Figures 2e, f**). Histological examination using Hematoxylin and Eosin (H&E) staining revealed that mice treated with dextran sulfate sodium (DSS) exhibited a loss of crypt structures, infiltration of mononuclear cells, and pronounced mucosal damage. In contrast, the group treated with AS-IV displayed reduced inflammatory cell infiltration, relatively preserved colonic architecture, and diminished mucosal damage ((**Figure 2g**). These results indicate that the administration of AS-IV significantly alleviated the pathogenic symptoms associated with DSS-induced ulcerative colitis (UC).

### 3.2 Gut microbiota analysis upon AS-IV treatment

**Figure 3:**
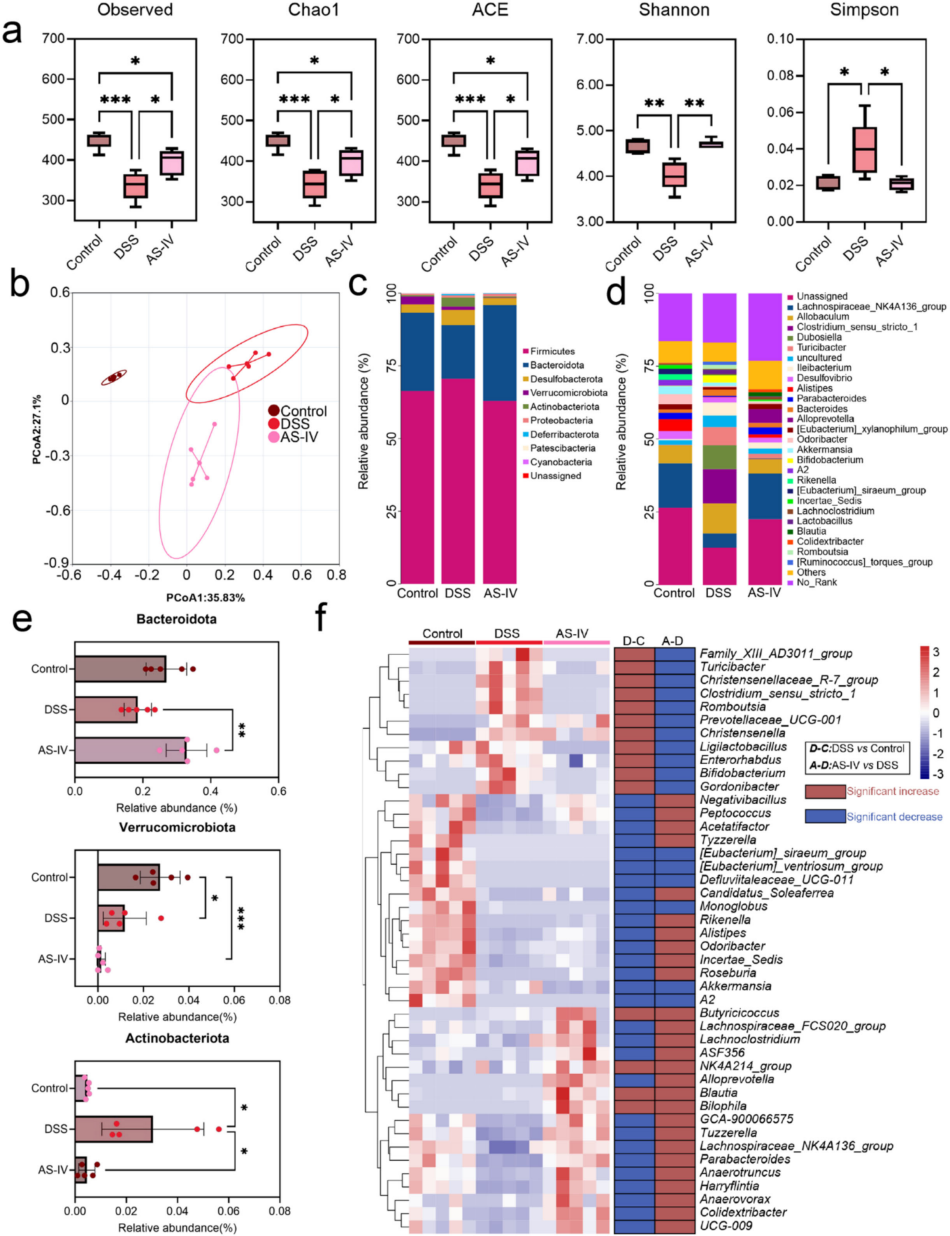
Gut microbiota analysis upon AS-IV treatment. **(a)** Alpha diversity boxplot.**(b)** Principal Co-ordinates Analysis (PCoA) of gut microbiota. **(c)** Taxonomic composition at the phylum level. **(d)** Taxonomic composition at the genus level. **(e)** Differential bacterial phylum level. **(f)** Heatmap of Genus-level differential bacterial.*P < 0.05; **P < 0.01; ***P < 0.001;.

The results presented above suggest that a reduction in gut microbiota diversity may contribute to the development of chronic inflammation, thereby necessitating strategies to regulate microbiota diversity and metabolism. In recent years, traditional Chinese medicine has gained attention due to its low side effect profile. Consequently, we employed AS-IV at a dosage of 15 mg/kg as an intervention strategy. Analysis of the gut microbiota revealed that AS-IV significantly mitigated the decline in alpha diversity, as evidenced by the alpha diversity indices Observed, Chao1, Simpson, Shannon, and ACE (**Figure 3A**). Furthermore, Principal Coordinates Analysis (PCoA) demonstrated significant separation among the three groups, indicating notable differences in microbial community composition (**Figure 3b**). The taxonomic landscapes of these groups exhibited similar communities at both the phylum and genus levels, with relatively high abundances of the *Lachnospiraceae-NK4A136 group*, *Allobaculum*, and the *Clostridium_sensu_stricto_1*. Moreover, it is apparent that AS-IV intervention has the potential to alleviate the reduction of the *Lachnospiraceae-NK4A136* group associated with chronic inflammation, as well as the proliferation of *Clostridium_sensu_stricto_1*(**Figures 3c, d**). The phylum-level analysis of differential bacterial populations indicated that AS-IV significantly mitigates the reduction in Bacteroidota and the increase in Actinobacteria induced by DSS (**Figure 3e**). At the genus level, the analysis demonstrated that AS-IV significantly upregulated 11 bacterial genera associated with chronic inflammation, including *Turicibacter*, *Clostridium sensu stricto 1*, and *Romboutsia*, while significantly downregulating 18 bacterial genera, such as *Peptococcus*, *Roseburi*a, and *Alistipes* (**Figure 3f**). Thereby, AS-IV treatment ameliorated disease progression and was characterized by the restoration of gut microbiota and metabolic product diversity.

### 3.3 AS-IV eases chronic enteritis by modulating microbial metabolism

**Figure 4:**
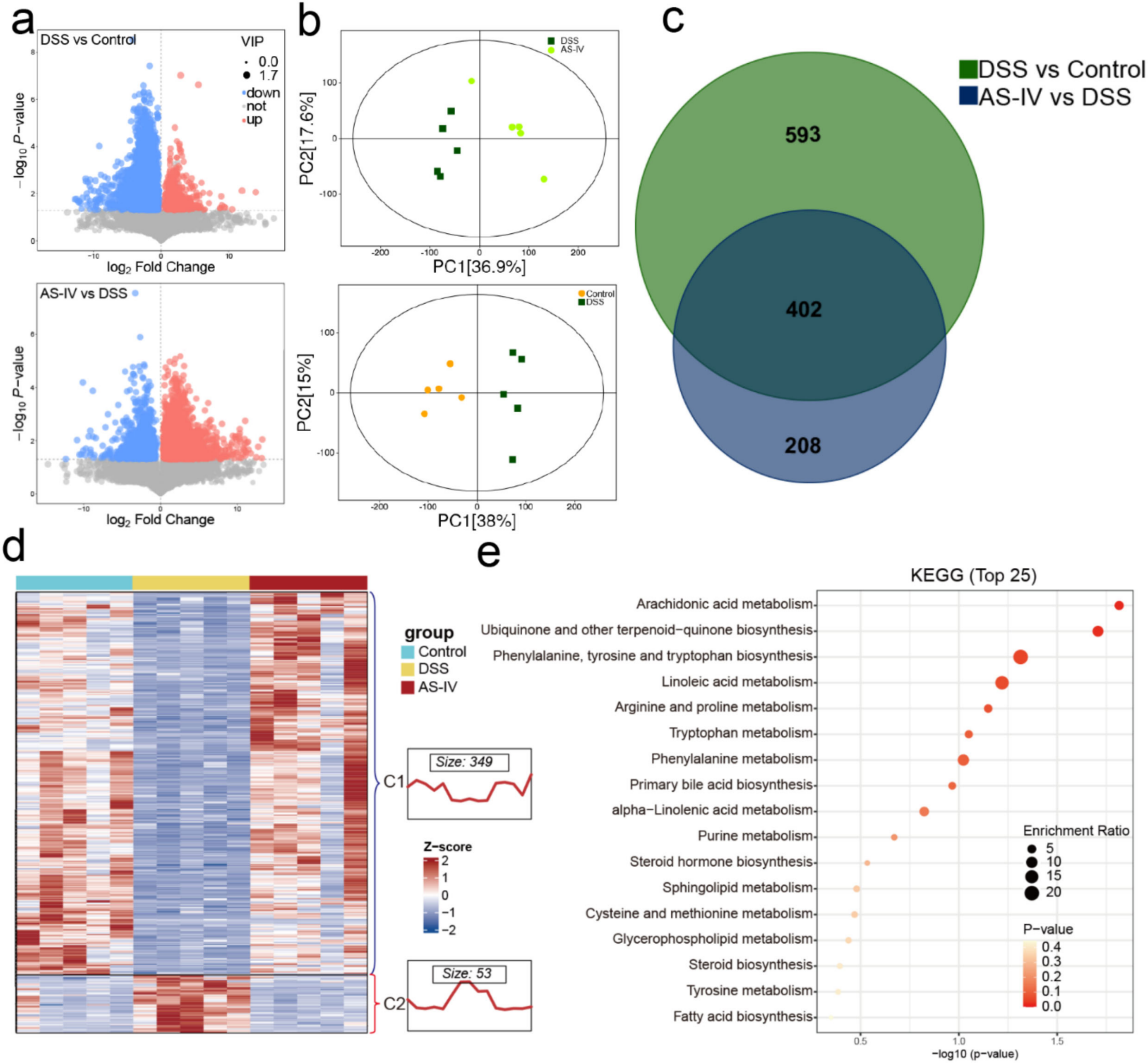
Metabolome analysis upon AS-IV treatment. **(a)** Volcano plot of differential metabolites. **(b)** Principal Co-ordinates (PCoA) Analysis of metabolites. **(c)** Venn diagrams of differential metabolites. **(d)** Hierarchical Clustering of differential metabolites. **(e)** KEGG analysis of differential metabolites.

Subsequently, we performed untargeted metabolomics analysis on animal models administered with AS-IV. The findings revealed that, in comparison to the Control group, the DSS group exhibited a total of 955 differential metabolites (with a VIP score greater than 1 and a FDR less than 0.05), of which 80 were upregulated and 875 were downregulated. In contrast, the AS-IV intervention group, when compared to the DSS group, demonstrated a total of 610 differential metabolites, with 494 upregulated and 116 downregulated (**Figure 4a**). Principal Coordinates Analysis (PCoA) indicated distinct clustering of metabolites among the Control, DSS, and AS-IV groups, signifying substantial separation among these groups (**Figure 4b**). Furthermore, a cross-analysis of metabolites modulated by AS-IV treatment in conjunction with CRC and CK treatment with CRC revealed that 349 metabolites were upregulated, whereas only 53 were downregulated (**Figures 4c, d**; **Supplementary Table S1**). Enrichment analysis of these differential metabolites reveals associations with several metabolic pathways, including arachidonic acid metabolism, ubiquinone and other terpenoid-quinone biosynthesis, phenylalanine, tyrosine, and tryptophan biosynthesis, linoleic acid metabolism, arginine and proline metabolism, tryptophan metabolism, phenylalanine metabolism, primary bile acid biosynthesis, alpha-linolenic acid metabolism, purine metabolism, steroid hormone biosynthesis, sphingolipid metabolism, cysteine and methionine metabolism, glycerophospholipid metabolism, steroid biosynthesis, tyrosine metabolism, and fatty acid biosynthesis, among others (**Figure 4e**). AS-IV treatment was associated with restoration of the gut microbiota and attenuation of inflammation, suggesting a potential role of microbiota modulation in its therapeutic effects.

### 3.4 Integrated study of microbiota and metabolome

**Figure 5:**
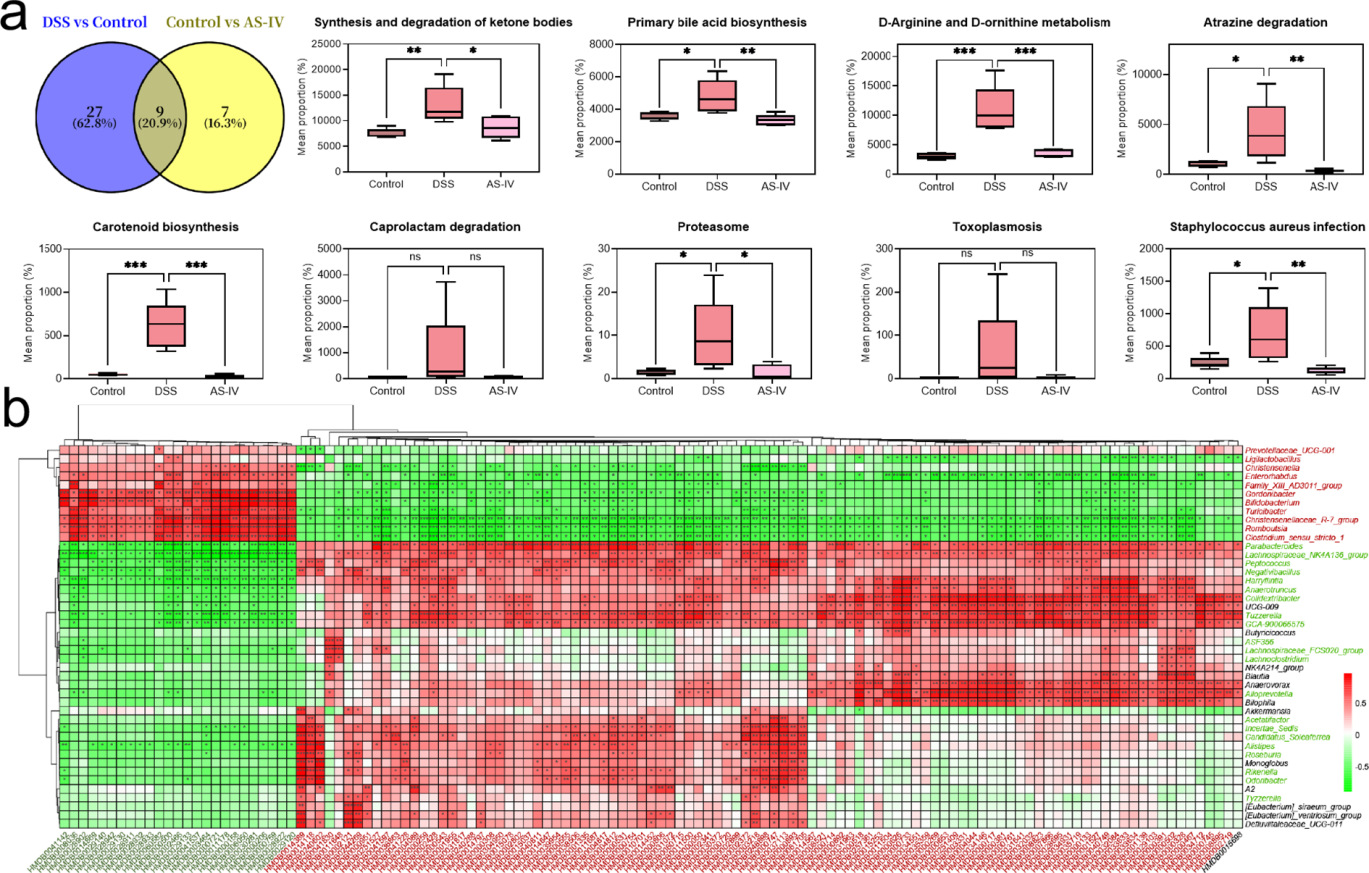
Integrative analysis of gut microbiota and metabolome reveals AS-IV-mediated regulatory networks. **(a)** Predicted functional pathways altered by AS-IV treatment based on PICRUSt2 analysis. **(b)** Heatmap showing Pearson correlation coefficients between differential gut microbiota taxa and differential metabolites.

Initially, we employed the PICRUSt2 analysis tool to predict and examine species functions based on amplicon sequencing data. The findings indicated that AS-IV could significantly mitigate pathways with elevated abundance in the DSS group, such as the synthesis and degradation of ketone bodies, primary bile acid biosynthesis, D-arginine and D-ornithine metabolism, atrazine degradation, carotenoid biosynthesis, caprolactam degradation, proteasome, toxoplasmosis, and *Staphylococcus aureus* infection. Upon comparing the differential metabolite enrichment pathways regulated by AS-IV, it was observed that only the primary bile acid biosynthesis pathway overlapped. This suggests that AS-IV may alleviate chronic inflammation by modulating the gut microbiota, thereby influencing the biosynthesis of primary bile acids (**Figure 5a**). Subsequently, a correlation analysis was performed on the differential microbiota and metabolism. The analysis revealed that in the DSS group, bacterial genera such as *Prevotellaceae-UCG-001*, *Clostridium sensu stricto 1*, and *Romboutsia*, which exhibited a significant increase but subsequently decreased following AS-IV intervention, demonstrated a significant negative correlation with metabolites that increased (and significantly decreased post-AS-IV intervention). Conversely, these genera showed a significant positive correlation with metabolites that decreased significantly (and increased after AS-IV intervention). In contrast, bacterial genera such as *Alistipes*, *Odoribacter*, and *Lachnoclostridium*, which significantly decreased in the DSS group and increased following AS-IV intervention, exhibited the opposite correlation trend (**Figure 5a**).

## 4 Discussion

Chronic inflammatory bowel diseases, such as ulcerative colitis, characterized by their persistent and recurrent nature, significantly impair the quality of life for millions of patients globally (Ungaro et al., 2017). Current clinical management predominantly depends on conventional pharmacological agents, including aminosalicylic acid derivatives, glucocorticoids, and immunosuppressants. While these medications effectively mitigate inflammation in the short term, their prolonged use is frequently associated with substantial adverse effects and fails to address the underlying issues of intestinal mucosal barrier restoration and microbial ecological reconstitution (Kaenkumchorn and Wahbeh, 2020; Porter et al., 2020; Segal et al., 2021). This study employed the traditional Chinese medicine monomer, AS-IV, to intervene in a dextran sulfate sodium (DSS)-induced chronic colitis model. By conducting comprehensive analyses of gut microbiota and untargeted metabolomics, the research delved into the therapeutic mechanisms, offering novel insights and a theoretical foundation for the clinical management of chronic colitis.

Traditional Chinese medicine monomers exhibit distinct advantages in the treatment of chronic enteritis, primarily due to their multi-target mechanisms of action and high safety profile (Shen and Yin, 2021; Li W. et al., 2023; Liu et al., 2025). AS-IV, a principal active component of the traditional Chinese medicine Huangqi (Astragalus membranaceus), not only retains the traditional therapeutic benefits of Huangqi—such as nourishing the middle and qi, eliminating decay, and promoting muscle growth—but also provides precise intervention due to its well-defined chemical structure (Liang Y. et al., 2023). Contemporary pharmacological research has demonstrated that AS-IV possesses a range of biological activities, including anti-inflammatory effects, immune regulation, and wound healing promotion (Tian et al., 2021; Gao et al., 2023; Zhang et al., 2023). In the context of ulcerative colitis treatment, its primary advantage is its capacity to simultaneously regulate intestinal microbiota balance, repair the intestinal mucosal barrier, and modulate the intestinal immune response, thereby creating a comprehensive therapeutic effect. This multi-pathway synergistic mechanism is challenging to replicate with conventional single-target drugs and mitigates the risk of drug interactions commonly associated with combination therapies (Zhu et al., 2022; Packer, 2023). In this study, a dosage of 15 mg/kg of AS-IV was administered to address DSS-induced chronic enteritis. The findings indicated that AS-IV effectively mitigated disease symptoms by modulating parameters such as weight gain, bleeding index, diarrhea index, and colon length.

A bidirectional regulatory relationship exists between the onset of chronic enteritis and the disruption of gut microbiota (Di Vincenzo et al., 2024). Intestinal inflammation can modify microbial composition, resulting in a reduction of beneficial bacteria and an abnormal proliferation of opportunistic pathogens. Conversely, microbiota dysbiosis can further compromise the integrity of the mucus layer, increase intestinal permeability, facilitate the translocation of pathogenic microorganisms and their metabolites, activate the mucosal immune system, and perpetuate a vicious cycle (Ni et al., 2017; Chen et al., 2021). The microbiota analysis conducted in this study corroborated this mechanism, demonstrating a significant decrease in microbiota diversity and an abnormal enrichment of pro-inflammatory bacteria in the DSS-induced chronic enteritis model. Notably, this dysbiosis is accompanied by barrier dysfunction, characterized by a reduction in goblet cell numbers and decreased expression of tight junction proteins within the intestinal mucosa (Bellaguarda and Chang, 2015). These factors collectively contribute to the pathological foundation for the persistence of chronic enteritis. The regulatory influence of AS-IV on gut microbiota is evident at multiple levels, as it significantly restores microbial diversity and enhances the relative abundance of beneficial bacteria, a finding corroborated by our research (Liu et al., 2024). Furthermore, this study identified that AS-IV modulates certain probiotics, including *Alistipes* and *Alloprevotella*, to mitigate the inflammatory process.

Omics analysis indicates that AS-IV can significantly modify the gut metabolic profile, notably enhancing the production of metabolites with anti-inflammatory properties (Han et al., 2025). These metabolites function as crucial signaling molecules, mitigating intestinal inflammation by inhibiting inflammatory pathways, such as the arachidonic acid pathway. Concurrently, the increased presence of flavonoids following AS-IV intervention warrants attention due to their antioxidant and immune-regulatory properties, which are particularly significant for ameliorating age-related changes in enteritis. These findings align with the results of this study. Furthermore, our investigation revealed that the integration of microbiota function prediction and metabolomics analysis demonstrates that AS-IV can modulate the synthesis pathway of primary bile acids to alleviate inflammation, suggesting a potential avenue for future research. The combined outcomes of metabolomics and microbiota analysis elucidate a critical mechanism: AS-IV may facilitate systemic regulation of chronic enteritis by reshaping the “microbiota-metabolite-host immune” axis. It should be noted that the sample size while appropriate for the initial histological and phenotypic assessments, represents a limitation for the exploratory metabolomics analysis, in which a large number of metabolites were screened. This relatively small sample size may limit the statistical power and the generalizability of the metabolomic findings. Nevertheless, the identified significant alterations in key metabolic pathways offer valuable preliminary insights and generate testable hypotheses for future validation.

From the perspective of translational medicine, AS-IV treatment for chronic enteritis presents significant clinical application potential. Unlike newer pharmaceuticals such as biologics, AS-IV, derived from traditional Chinese medicine, offers advantages in terms of safety, cost-effectiveness, and suitability for long-term use. In the post-antibiotic era, there is a growing emphasis on regulatory strategies that target the gut microbiota (Boccellino, 2023). AS-IV mitigates the risk of microbial dysbiosis associated with traditional antibiotics by restoring microbial balance rather than merely eradicating bacteria. Based on the findings of this study, future research could explore several directions: firstly, the development of individualized AS-IV administration strategies tailored to patients’ microbiota profiles and metabolic phenotypes to achieve precise interventions; secondly, the formulation of synbiotics incorporating AS-IV, potentially in combination with prebiotics such as oligogalactose, to enhance the synergistic promotion of specific probiotics, such as *Lactobacillus reuteri*. The third objective is to investigate the combined therapeutic approach of AS-IV and conventional Western medicine, leveraging its benefits in mucosal repair and microecological regulation to address the limitations of current anti-inflammatory drugs and enhance overall efficacy.

AS-IV, an active monomer extracted from traditional Chinese medicine, has exhibited distinct advantages in the treatment of chronic enteritis through its multi-target and multi-level mechanisms of action (Wang and Wang, 2021; Botelho et al., 2023). Its primary value lies in its capacity to synergistically regulate gut microbiota balance, facilitate mucosal barrier repair, and reestablish gut immune homeostasis, thereby creating a comprehensive therapeutic effect. This integrated regulatory impact not only embodies the holistic therapeutic principles of traditional Chinese medicine but also aligns with the developmental trajectory of contemporary microecological medicine. As research on gut microbiota and metabolomics advances, AS-IV is anticipated to become a significant option for the comprehensive management of chronic enteritis, offering novel solutions to enhance patients’ quality of life.

## 5 Conclusions

This study demonstrated the substantial protective effect of AS-IV in mitigating chronic enteritis, as exemplified by the DSS-induced colitis model utilized herein. Furthermore, the research delved into the underlying mechanisms, which are intricately linked to the composition of the gut microbiota and its metabolites. These findings offer a novel perspective on the pharmacological actions of AS-IV and underscore the pivotal role of gut microbiota in modulating the anti-inflammatory effects of monomers derived from traditional Chinese medicine.

## Data Availability

The metabolomics data presented in this study have been deposited in the OMIX database of the China National Center for Bioinformation / Beijing Institute of Genomics, Chinese Academy of Sciences (accessible at https://ngdc.cncb.ac.cn/omix) under the accession number OMIX 010518. The 16S rRNA gene sequences are available under the accession number CRA026681.
